# Mapping the microbial diversity associated with different geochemical regimes in the shallow-water hydrothermal vents of the Aeolian archipelago, Italy

**DOI:** 10.3389/fmicb.2023.1134114

**Published:** 2023-08-11

**Authors:** Bernardo Barosa, Alessandra Ferrillo, Matteo Selci, Marco Giardina, Alessia Bastianoni, Monica Correggia, Luciano di Iorio, Giulia Bernardi, Martina Cascone, Rosaria Capuozzo, Michele Intoccia, Roy Price, Costantino Vetriani, Angelina Cordone, Donato Giovannelli

**Affiliations:** ^1^Department of Biology, University of Naples “Federico II”, Naples, Italy; ^2^Blue Marine Foundation, London, United Kingdom; ^3^School of Marine and Atmospheric Sciences, Stony Brook, NY, United States; ^4^Department of Biochemistry and Microbiology, Rutgers University, New Brunswick, NJ, United States; ^5^Department of Marine and Coastal Science, Rutgers University, New Brunswick, NJ, United States; ^6^Istituto per le Risorse Biologiche e Biotecnologiche Marine, Consiglio Nazionale Delle Ricerche, CNR-IRBIM, Ancona, Italy; ^7^Earth-Life Science Institute, Tokyo Institute of Technology, Ookayama, Tokyo, Japan; ^8^Marine Chemistry and Geochemistry Department–Woods Hole Oceanographic Institution, Woods Hole, MA, United States

**Keywords:** shallow-water vents, Aeolian archipelago, 16S rRNA amplicon sequencing, microbial diversity, marine protected areas, hydrothermal vents

## Abstract

Shallow-water hydrothermal vents are unique marine environments ubiquitous along the coast of volcanically active regions of the planet. In contrast to their deep-sea counterparts, primary production at shallow-water vents relies on both photoautotrophy and chemoautotrophy. Such processes are supported by a range of geochemical regimes driven by different geological settings. The Aeolian archipelago, located in the southern Tyrrhenian sea, is characterized by intense hydrothermal activity and harbors some of the best sampled shallow-water vents of the Mediterranean Sea. Despite this, the correlation between microbial diversity, geochemical regimes and geological settings of the different volcanic islands of the archipelago is largely unknown. Here, we report the microbial diversity associated with six distinct shallow-water hydrothermal vents of the Aeolian Islands using a combination of 16S rRNA amplicon sequencing along with physicochemical and geochemical measurements. Samples were collected from biofilms, fluids and sediments from shallow vents on the islands of Lipari, Panarea, Salina, and Vulcano. Two new shallow vent locations are described here for the first time. Our results show the presence of diverse microbial communities consistent in their composition with the local geochemical regimes. The shallow water vents of the Aeolian Islands harbor highly diverse microbial community and should be included in future conservation efforts.

## 1. Introduction

The vast and diverse metabolic repertoire of microorganisms enables their ubiquitous distribution on our planet, making them key mediators of biogeochemical cycles on a planetary scale (Falkowski et al., 2008). Notwithstanding their ubiquity, their distribution and the environmental factors shaping it, are not fully resolved. However, significant correlations between microbial community composition and environmental geochemistry have been established in diverse environments (Jorgensen et al., 2012; Alsop et al., 2014; Liu et al., 2014; Yamamoto et al., 2019). Geothermal environments are ideal systems to study the complex relationships between community composition and geochemistry due to the wide geochemical gradients present across proximal geographic distances, and relatively constrained microbial communities (Power et al., 2018). Additionally, geothermal ecosystems harbor diverse communities of extremophiles that are an untapped source of potential biotechnological compounds (Sayed et al., 2020). Hence, there is an increasing necessity to characterize in detail the microbial diversity in these ecosystems and to prioritize their conservation.

Marine hydrothermal vents are dynamic systems where water is discharged through the Earth’s crust in areas where there is enough heat to drive fluid circulation, e.g., plate boundaries and mid-ocean ridges (Price and Giovannelli, 2017; Brovarone et al., 2020). The interaction between the seawater with the hot, deep-subsurface lithologies, promotes the leaching of inorganic reduced species, such as volatiles and metals (McDermott et al., 2018). Upon arrival at the surface, the mixing of the reduced hydrothermal fluids (enriched in electron donors) with the oxidized sea water (enriched in electron acceptors), creates a thermodynamic disequilibrium that fuels diverse microbial consortia and sustains complex food webs (Dahle et al., 2015; Price et al., 2015; Reed et al., 2015). In contrast with their deep-sea counterparts, shallow-water hydrothermal vents (SWHV) are closer to the surface, thus they are strongly influenced by solar energy. In these shallow systems, the primary production is reliant upon a mixture of phototrophy and chemolithotrophy, making them high-energy environments (Tarasov et al., 2005; Gomez-Saez et al., 2017). Additionally, the shallower depths and the resultant decrease in pressure allow for the presence of a generally abundant free gas phase (Bastianoni et al., 2022). The proximity to landmasses promotes the input of terrigenous organic carbon to the system, while tidal waves and tides make SWHV transient ecosystems subject to strong dynamic forcing (Giovannelli et al., 2013; Yücel et al., 2013; Price and Giovannelli, 2017).

The shallow-water hydrothermal vents of the Aeolian archipelago are some of the most studied marine shallow vents in the world. The archipelago, located north of Sicily, is composed of seven volcanic islands (Stromboli, Vulcano, Panarea, Salina, Lipari, Alicudi, and Filicudi) and several sea-mounts. The archipelago lies between the Tyrrhenian sea back-arc basin and the Calabrian fore-arc. While most of its volcanic activity developed during the Quaternary period, it continues to the present day on some of the islands (Vulcano and Stromboli) in the form of volcanic eruptions and secondary geothermal manifestations (Gamberi et al., 1997). The geomorphism of the region is very dynamic, marked by the presence of rock types belonging mainly to the calc-alkaline, shoshonitic, and potassium associations (De Astis et al., 2000; Peccerillo and Turco, 2004). The islands of Lipari, Salina, and Vulcano, are aligned on an NNW–SSE striking fault, while a NNE–SSW to NE–SW striking fault affects the islands of Stromboli and Panarea (De Astis et al., 2003; Favallim et al., 2005). Most of the described marine geothermal activity is present along the coast of the islands of Vulcano and Panarea, where the majority of microbiological studies on the shallow-water vents have been focused so far. Despite this, additional sites of geothermal activities have been described on the other islands (Cardellini et al., 2003; Inguaggiato et al., 2012, 2013).

Previous studies have mapped the thermodynamic chemical reaction landscape of SWHVs of Vulcano and Panarea islands, showing that a wide range of exergonic chemical reactions are capable of sustaining diverse metabolic strategies (Amend et al., 2003; Rogers and Amend, 2005, 2006; Rogers et al., 2007; Price et al., 2015). Accordingly, through metagenomics analysis, amplicon sequencing, and DGGE analysis, previous studies reported complex and diverse microbial communities inhabiting these ecosystems (Gugliandolo and Italiano, 1999; Simmons and Norris, 2002; Rogers and Amend, 2005, 2006; Rusch et al., 2005; Lentini et al., 2007, 2014; Manini et al., 2008; White et al., 2008; Maugeri et al., 2009, 2010, 2013; Gugliandolo et al., 2012, 2015; Placido et al., 2015; Antranikian et al., 2017; Fagorzi et al., 2019; Gugliandolo and Maugeri, 2019; Sciutteri et al., 2022). These communities were found to be enriched in sulfur-oxidizing chemolithoautotrophic bacteria at higher temperatures and at lower temperatures by heterotrophic and photoautotrophic-based life-styles. Furthermore, thermophilic microorganisms have been isolated from several vents (Hafenbradl et al., 1996; Deckert et al., 1998; Lentini et al., 2007; Gugliandolo et al., 2012). However, only a few studies tried to couple in more detail both the geochemistry of the shallow vents and the microbial communities inhabiting them. In addition, these studies were conducted exclusively on the SWHV of Vulcano and Panarea, with the exception of one study, based on culture-dependent approaches that included communities from Lipari and Stromboli (Gugliandolo and Italiano, 1999). Currently, there is a lack of a more comprehensive survey of the microbial communities associated with the diversity of shallow-water hydrothermal vent ecosystems of the Aeolian archipelago.

To address this, and to understand how geochemically different shallow-water hydrothermal vents shape the microbial community composition, we used a combination of 16S rRNA gene amplicon sequencing and geochemical approaches to survey six shallow-water hydrothermal vents located in four different islands of the Archipelago, including two new locations that have never been characterized in the literature before. Our results show that shallow-water hydrothermal vents of the Aeolian islands are ecosystems that support diverse and complex microbial communities, providing important ecological roles to the surrounding marine environment. For this reason, they should be included in current and future conservation efforts.

## 2. Materials and methods

### 2.1. Sampling and site description

**Figure 1:**
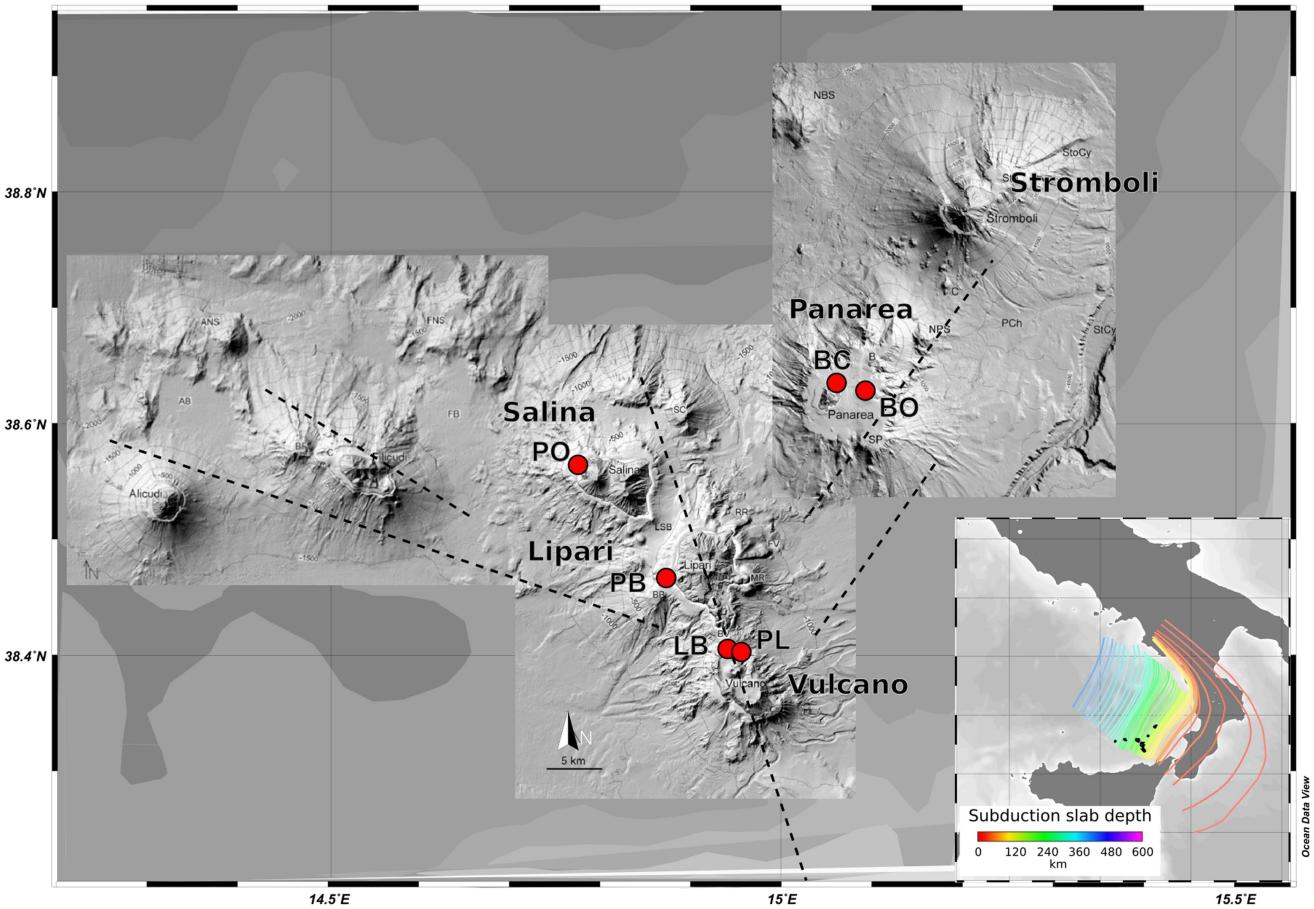
Map of the volcanic Aeolian island archipelago, located north of Sicily. Sample sites are shown in red. Major fault systems are illustrated here, as well as the gradient of the different slab depths (100–250 Km).

The samples were retrieved on the AEO19 expedition to the islands of the Aeolian archipelago: Vulcano, Panarea, Salina, and Lipari in the summer of 2019. Six different locations were sampled: Vulcano - Levante Bay (LB), Vulcano - Levante Port (PL); Panarea - Bottaro (BO), Panarea - Baia Calcara (BC), Salina - Pollara (PO), and Lipari - Pietra del bagno (PB) (Figure 1), representing different shallow-water geothermal environments. All the samples were retrieved by SCUBA divers, using sterile falcon tubes and syringes. To accomplish a broader characterization of the different sites, different types of samples were collected: sediments, hydrothermal fluids, and biofilms. In the sites Levante Bay, Pollara, Baia Calcara, and Pietra del Bagno, abundant white microbial mats were observed near the venting sites. The shallow vents across the sampled locations were sulfide-rich, with the exception of the Pietra del Bagno site, where the presence of conspicuous orange precipitates suggests the presence of iron-rich hydrothermal fluids. Intense degassing was observed in the Bottaro venting site. All samples were stored either at +4°C or − 20°C in accordance to the specific protocols for later processing in the lab. Subsamples used for molecular analysis and organic matter determination were stored frozen at −20°C, while samples for the determination of the major ions were filtered through a 0.22 μm filter and stored at +4°C. Temperature was measured *in situ* using a waterproof thermocouple, while pH, oxidation–reduction potential, conductivity, and dissolved oxygen were measured using a multi-parameter sensor probe (Hanna- multiparameter, model HI98194) on the boat immediately after the dive, with a fluid sample collected with syringes at the seafloor.

### 2.2. Sedimentary organic matter

The determination of the concentration of pigments (chlorophyll-*a* and phaeopigments) was carried out according to Lorenzen and Downs (1986). Briefly, the samples were supplemented with 90% of acetone and further incubated in the dark, at 4°C, for 12 h. After incubation, in order to remove the sediments, the samples were centrifuged and the concentration of chlorophyll-*a* and phaeopigments was estimated using a spectrofluorometer (at wavelengths of 430 nm and 630 nm), before and after being supplemented with 200 μL of 0.1 N HCl, respectively. Pigment concentrations were normalized to sediment dry weight (Manini et al., 2003).

Total protein concentrations were determined according to Hartree (1972), and further modified for analysis of sediment samples from Fabiano et al. (1995). The total carbohydrate concentrations were determined according to Gerchakov and Hatcher (1972), and expressed as glucose equivalents. Total lipids were extracted from the sediments by a direct elution with chloroform and methyl alcohol (1:1 v/v), and further determined according to Bligh and Dyer (1959). The readings were performed spectrophotometrically. The concentrations of carbohydrates, proteins and lipids were converted to carbon equivalents using the conversion factors, 0.40, 0.49 and 0.75 μg C × μg^−1^, respectively, and further normalized to sediment dry weight (Fabiano et al., 1995). The biopolymeric carbon (BPC), calculated through the sum of the concentration of carbohydrates, proteins and lipids, represents the fraction of the total organic carbon potentially available to heterotrophs and benthic consumers in the environment. All measurements are reported as an average of three replicates.

### 2.3. Geochemical analysis

The concentrations of major cations (sodium-Na^+^, potassium-K^+^, magnesium-Mg^2+^ and calcium-Ca^+^) and anions (chlorine-Cl^−^, sulfate-SO_4_^2−^ and bromide-Br^−^) of the hydrothermal fluids were measured using ion chromatography (ECO, IC Metrohm) equipped with conductivity detectors in two independent measurements. Calibration curves for the ion species of interest were run in the range of 0.1 and 10 ppm with *R* ≥ 0.999. In order to reduce the amount of Cl^−^ and Na^+^ entering the system, before the analysis, all samples were filtered (0.22 μm) and diluted to guarantee a conductivity of 600 μS/cm. All dilutions were made using 18.2 MΩ/cm type I water, which was also used as a blank for blank subtractions. Anions were run using a 3.2 mM Na_2_CO_3_ + 1 mM NaHCO_3_ mobile phase on a Metrosep A Supp 5 column equipped with a 0.15 M ortho-Phosphoric acid suppressor. The flow of the anionic eluent was 0.7 mL min-1 for 30 min, effective for good resolution of all anions in the standard solution. Cations were run using a 2.5 mM HNO_3_ + 0.5 mM (COOH)_2_ × 2H_2_O mobile phase on a Metrosep C4 column. The flow of the cationic eluent was 0.9 mL min-1 with a total separation of 35 min, effective for good resolution of all cations in the standard solution. Data acquisition and analysis were carried out through MagIC Net 3.3 software. Additionally, calibration curves were carried out using certified CPA chem external standards for each of the anions and cations analyzed. Detection limits for each of the ions analyzed correspond to 95% confidence levels, and were as follows: Mg^2+^ (0.08 mM), Na^+^ (0.3 mM), Ca^2+^ (0.3 mM), K^+^ (0.7 mM), SO_4_^2−^ (0.03 mM), Br^−^ (0.02 mM), Cl^−^ (0.1 mM).

### 2.4. DNA extraction and amplicon sequencing

DNA extraction was performed using the DNeasy PowerSoil Kit (QIAGEN), following the manufacturer’s instructions, with small modifications. The modifications of the protocol included a further elution step to better recover the DNA from the column. For samples where the PowerSoil kit protocol resulted in low quantity of DNA, a modified phenol-chloroform extraction method was employed (Cordone et al., 2005, 2011; Zanfardino et al., 2016), specifically adapted for the conditions found in shallow-water hydrothermal vents, such as the higher presence of clays and anions (Giovannelli et al., 2013, 2016). Briefly, 0.8 g of sediment was suspended in 850 μL of extraction buffer (100 mM Na_2_HPO_4_ pH 8, 100 mM tris–HCL pH 8, 100 mM EDTA pH 8, 1.5 M NaCl, 1% CTAB) and further supplemented with 100 μL of Lysozyme (100 mg/mL), followed by two incubation periods at 37°C for 30 min, the latter after the addition of 5 μL of Proteinase K (1 mg/mL). Subsequently, 50 μL of 20% SDS was added to the samples and incubated 1 h at 65°C, with regular mixing. The sediments were removed through consecutive centrifugations (2 × 14,000 g) after the addition of a solution of phenol:chloroform:isoamyl alcohol (25:24:1/24:1). The supernatant was collected and supplemented with Na-acetate (0.1 vol) and isopropanol 100%, and incubated at room temperature for 12 h. The precipitated DNA was then washed with 70% ice-cold ethanol, and re-suspended in 50 μL of Tris–HCL. DNA was visualized with agarose gel electrophoresis and quantified spectrophotometrically and spectrofluorimetrically (NanoDrop and Qubit). Obtained DNA was sequenced at the Integrated Microbiome Resource (IMR)^1^ using primers targeting the V4-V5 of the 16S rRNA (515FB = GTGYCAGCMGCCGCGGTAA 926R = CCGYCAATTYMTTTRAGTTT515FB), using Illumina MiSeq technology.

### 2.5. Bioinformatic and statistical analysis

The raw sequence data was processed using the DADA2 package (Callahan et al., 2017). The analysis of the quality profile was carried out after removing primers and adapters. Sequences with an average call quality for each base between 20 and 40, were retained for downstream analysis. DADA2, through estimation of the error profile for each possible transition, identifies amplicon sequencing variants (ASVs). Taxonomy was assigned to the ASVs by matching with the SILVA Database, release 138.1^2^ (Quast et al., 2013). Taxonomic assignments, together with the variant abundance table, were used to calculate diversity indices and investigate microbial diversity with the Phyloseq package (McMurdie and Holmes, 2013), as previously reported (Cordone et al., 2022). Briefly, the ASVs count table and the assigned taxonomy were combined into a *phyloseq* object. Mitochondria, Chloroplast, and Eukaryotic sequences were removed. Then, groups related to human pathogens and common DNA extraction contaminants (Sheik et al., 2018) were removed. The remaining reads represented 96.97% of the original reads, with 149,351 reads assigned to 2,145 ASVs preserved for downstream analysis. Diversity analyzes were carried out using the Phyloseq package (McMurdie and Holmes, 2013) with the relative abundance values set to 100%. Top abundance ASVs and genera were further defined as having accumulative abundances above 0.1%. Additionally, the alpha diversity was investigated using Shannon diversity index. The beta diversity was investigated using the UniFrac diversity index (Unweighted and Weighted) as implemented in the vegan package (Oksanen et al., 2018). Subsequently, the resultant similarity matrix was plotted using non-metric multidimensional scaling techniques. The resulting ordination was used to investigate the correlation between the environmental and geochemical variables using environmental fitting (*envfit* and *ordisurf* functions in vegan). All statistical investigations, data processing and plotting were carried out in the R statistical software version 4.1.2 (R Core Team, 2021), using the vegan (Oksanen et al., 2018), ggplot2 (Wickham, 2011), and the packages supra-referred. All the sequences analyzed in this study are available through the European Nucleotide Archive (ENA) under project accession PRJEB54611 under the ENA Umbrella project CoEvolve PRJEB55081. A complete R script containing all the steps to reproduce our analysis is available at https://github.com/giovannellilab/Barosa_et_al_Aeolian_shallow_vent_diversity with DOI: https://doi.org/10.5281/zenodo.7480614 together with all the environmental and geochemical data.

## 3. Results

### 3.1. Physico-chemical parameters and sedimentary organic matter content

The physico-chemical parameters of the hydrothermal sites sampled in the present study are presented in Table 1. Overall, the physico-chemical parameters measured varied between the sites. The shallow-water vent sediment temperatures at the sampled venting orifice ranged from 28°C in Pollara to 90°C in Baia Calcara. The salinity ranged from 33.0 psu in Levante Bay to 41.5 psu in Pietra del Bagno. Dissolved oxygen (DO) values ranged from 11% in Levante Bay to 57.3% in Pietra del Bagno. Additionally, the oxidation–reduction potential of the different sites was negative for Levante Bay, Bottaro, and Levante Port, while Baia Calcara, Bottaro, and Pietra del Bagno had positive reduction potentials. The pH was slightly acidic for every sample, with the exception of Bottaro, which had circumneutral pH (8).

**Table 1:** Location (GPS coordinates), depth, and the physico-chemical parameters measured on the venting fluids at the different sampled locations.

Station	Island	Latitude (°N)	Longitude (°E)	Depth (m)	Temperature (°C)	Salinity (PSU)	DO (%)	ORP (mV)	pH
LB	Vulcano	38.416729	14.959821	0.2	83.9	33.0	11	−295	5.8
PL	Vulcano	38.4161941	14.9611976	6.1	52	37.7	24.4	−202	5.22
BC	Panarea	38.64557	15.076182	15	90	37.5	43	150	5.26
BO	Panarea	38.6388904	15.1104468	6.5	46	40.94	15	−260	5.4
PO	Salina	38.574516	14.799716	10	28	39.0	48	121	5.6
PB	Lipari	38.47514	14.898117	12	45	41.5*	57.3	77	8.08

ORP - Oxido-reduction potential; DO - Dissolved oxygen; *Value of salinity for Pietra del Bagno calculated from Cl− concentrations using the formula: Salinity (ppt) = 0.00180665 × Cl− (mg/L).

**Figure 2:**
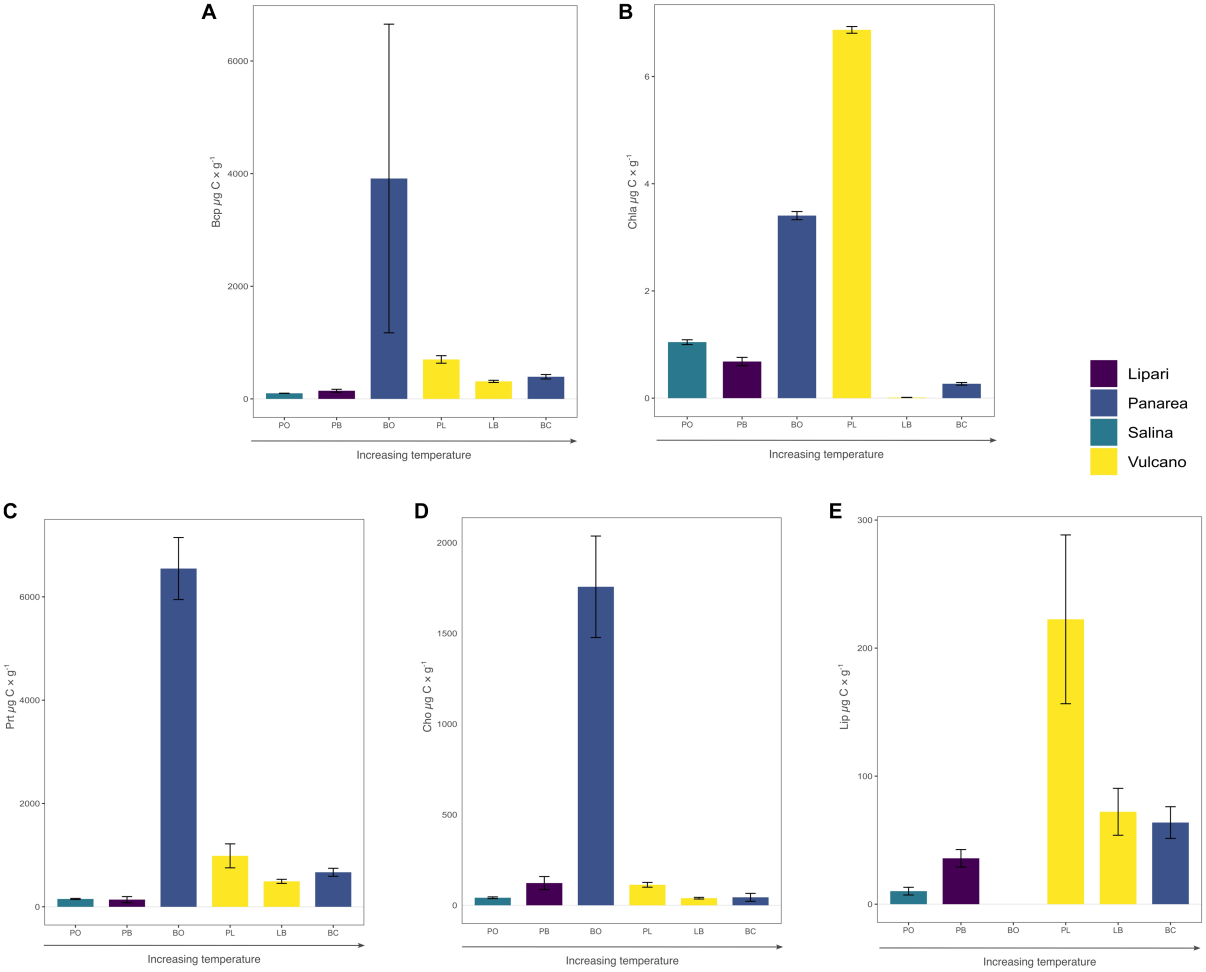
Content and quality of the organic matter measured in the sampled sediments. **(A)** Biopolymeric organic carbon (BCP); **(B)** Chlorophyll-a pigments content; **(C)** Proteins; **(D)** Carbohydrates; **(E)** Lipids. All sites are organized by increasing temperatures and are expressed in μg g^−1^ of dry sediment. Error bars represent standard deviation (*n* = 3).

The sampled sediments showed a wide range of sedimentary organic matter content, with concentrations of biopolymeric organic carbon (BPC) ranging from 99.44 μg C g^−1^ (± 2.22) in Pollara to 3499.0 μg C g^−1^ (± 2740.5) in Bottaro. The higher values of BPC in Bottaro, as well as the high variability between the measured replicates, was hypothesized to be related to the presence of a biofilm ingrained in the coarse sediment. Total protein and carbohydrates ranged from 492.88 μg C g^−1^ (± 39.5) and 39.23 μg C g^−1^ (± 4.64) in Levante Bay to 6547.72 μg C g^−1^ (± 599.9) and 1756.92 μg C g^−1^ (± 279.88) in Bottaro, respectively. Total lipids ranged from 10.17 μgC g^−1^ (± 3.02) in Pollara to 222.48 μg C g-1 (± 65.91) in Levante Port. Protein to carbohydrate ratios ranged from 1.1 in Pietra del Bagno to 15.2 in Baia Calcara. Chlorophyll-*a* concentration values ranged from 0.01 μg g^−1^ (± 0.003) in Levante Bay to 6.9 μg g^−1^ (± 0.126) in Levante Port. The highest chlorophyll-*a* concentrations were measured at the moderate temperature sites, Bottaro and Levante Port, while the lowest values were measured at the high temperature sites, Levante Bay and Baia Calcara (Figure 2).

### 3.2. Geochemical analysis

**Figure 3:**
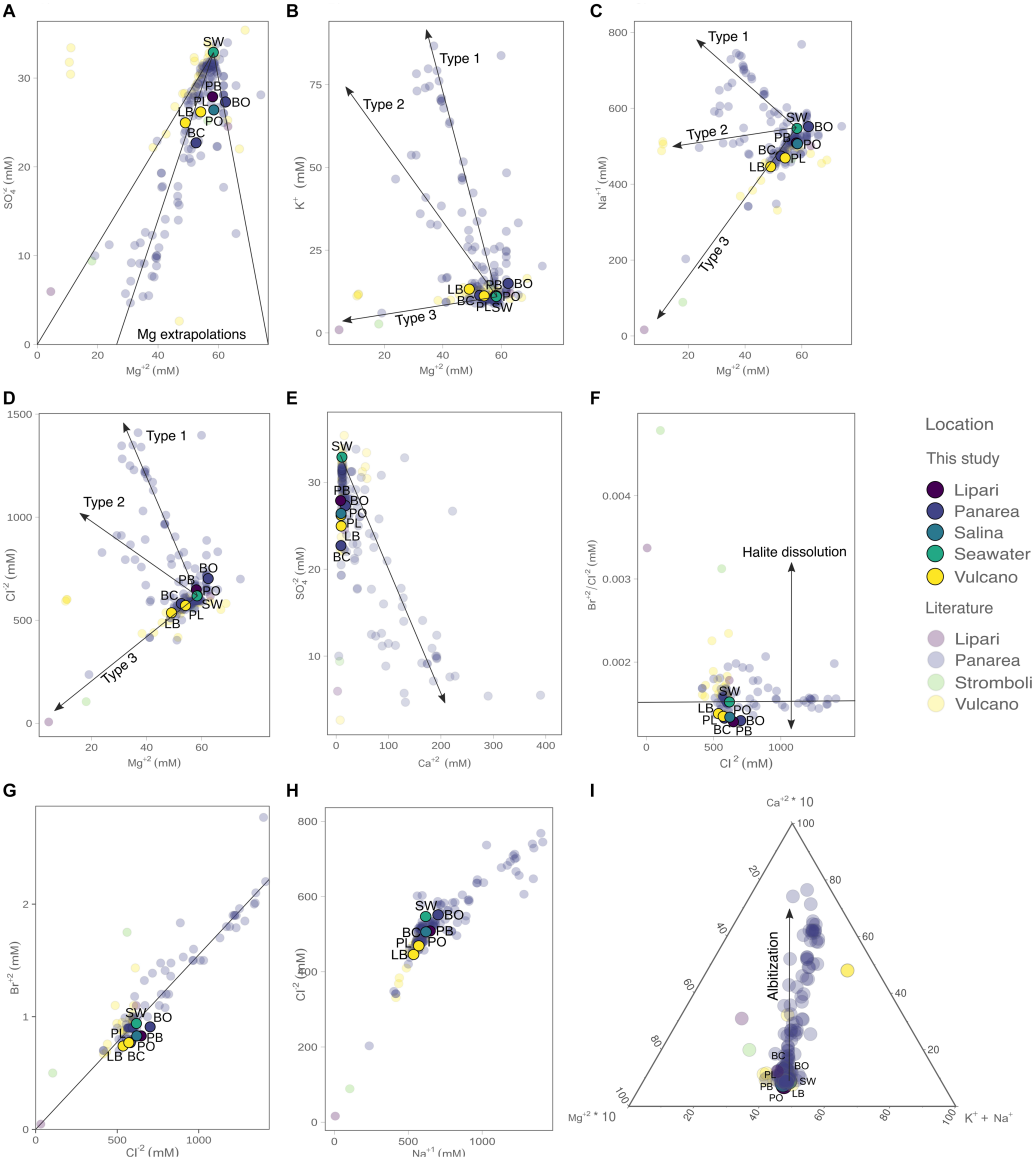
Concentrations of the major anions and cations measured in the present study (bold colors), as well as from previous reports from the Literature (opaque colors): **(A)** SO_4_^2−^ vs. Mg^2+^, with the three visible Mg^2+^ end-member extrapolations; **(B)** K^+^ vs. Mg^2+^ with the three fluid mixing trends observed; **(C)** Na^+^ vs. Mg^2+^; **(D)** Cl^−^ vs. Mg^2+^; **(E)** SO_4_^2−^ vs. Ca^2+^ (vector illustrating end-member extrapolation); **(F)** Br^−^/Cl^−^ ratio vs. Cl^−^, where it is possible to infer the presence of halite in solution; **(G)** Br^−^ vs. Cl^−^ and extrapolations to zero; **(H)** Cl^−^ vs. Na^+^; **(I)** ternary plot with the influence of albitization on the hydrothermal fluids. The literature data was retrieved from Italiano and Nuccio (1991), Sedwick and Stüben (1996), Gugliandolo et al. (2006), Rogers et al. (2007), Sieland (2009), Tassi et al. (2009), Inguaggiato et al. (2013), Price et al. (2015), and Kürzinger (2019).

The concentration of the major elements in the hydrothermal fluids were measured in the forms of major anions and cations (Figure 3; Table 2). Chloride concentrations ranged from 535.9 (% RDS 0.38) to 701.9 mM (% RDS 0.38), compared to 618.0 mM in seawater. Bottaro, Pollara and Pietra del Bagno have the highest Cl^−^ values. Sulfate concentrations ranged from 22.71 mM (% RDS 0.69) in Baia Calcara to 27.89 mM (% RDS 0.69) in Pietra del Bagno. Sulfate was depleted in the hydrothermal fluids of all the samples compared to seawater concentrations (32.9 mM). Additionally, Br^−^ concentrations ranged from 0.74 mM (% RDS 0.59) in Levante Bay to 0.91 mM (% RDS 0.59) in Bottaro, compared to sea water concentrations (0.94 mM). As for the major cations, Mg^2+^ concentrations ranged from 49.04 mM (% RDS 3.8) in Levante Bay to 62.36 mM (% RDS 3.8) in Bottaro, compared to seawater concentrations of 58.3 mM. Bottaro and Pollara were the only sites where fluids were enriched in Mg^2+^, relative to seawater. The concentrations of K^+^, Ca^2+^ and Na^+^ ranged from 11.18 mM (% RDS 11.75) in Pollara to 13.23 mM (% RDS 11.75) in Levante Bay, from 8.55 mM (% RDS 10.37) in Pietra del Bagno to 17.87 mM (% RDS 10.37) in Bottaro, and from 445.9 mM (% RDS 7.4) in Levante Port to 551.6 mM (% RDS 7.4) in Bottaro, compared to the seawater concentrations of 11.0 mM, 11.1 mM and 547.0 mM, respectively. Additionally, we combined our data from previous publish data on the geochemistry of the fluids sampled from the shallow water hydrothermal vents of Vulcano, Panarea, and Stromboli (Italiano and Nuccio, 1991; Sedwick and Stüben, 1996; Gugliandolo et al., 2006; Rogers et al., 2007; Sieland, 2009; Tassi et al., 2009; Inguaggiato et al., 2013; Price et al., 2015; Kürzinger, 2019). Fluid data from the present study falls within a mixing trend resembling Type 3 fluids previously described by Price et al., 2015.

**Table 2:** Concentrations of the major anions and cations (mM) of the hydrothermal fluids from the Aeolian Shallow-water vents.

Station	Island	Mg^2+^	Na^+^	Ca^2+^	K^+^	SO_4_^2−^	Br^−^	Cl^−^
LB	Vulcano	49.04	445.9	9.65	13.23	24.96	0.74	535.9
PL	Vulcano	54.13	469.1	9.47	11.19	26.19	0.77	571.2
BC	Panarea	52.6	474.1	9.25	11.37	22.71	0.77	580.6
BO	Panarea	62.36	551.6	17.87	14.96	27.31	0.91	701.9
PO	Salina	58.46	506.3	9.41	11.18	26.41	0.83	620.2
PB	Lipari	58.06	508.6	8.55	11.32	27.89	0.83	647.4
Sea Water	–	58.3	547.0	11.1	11.0	32.9	0.94	618.0

### 3.3. Microbial diversity

**Figure 4:**
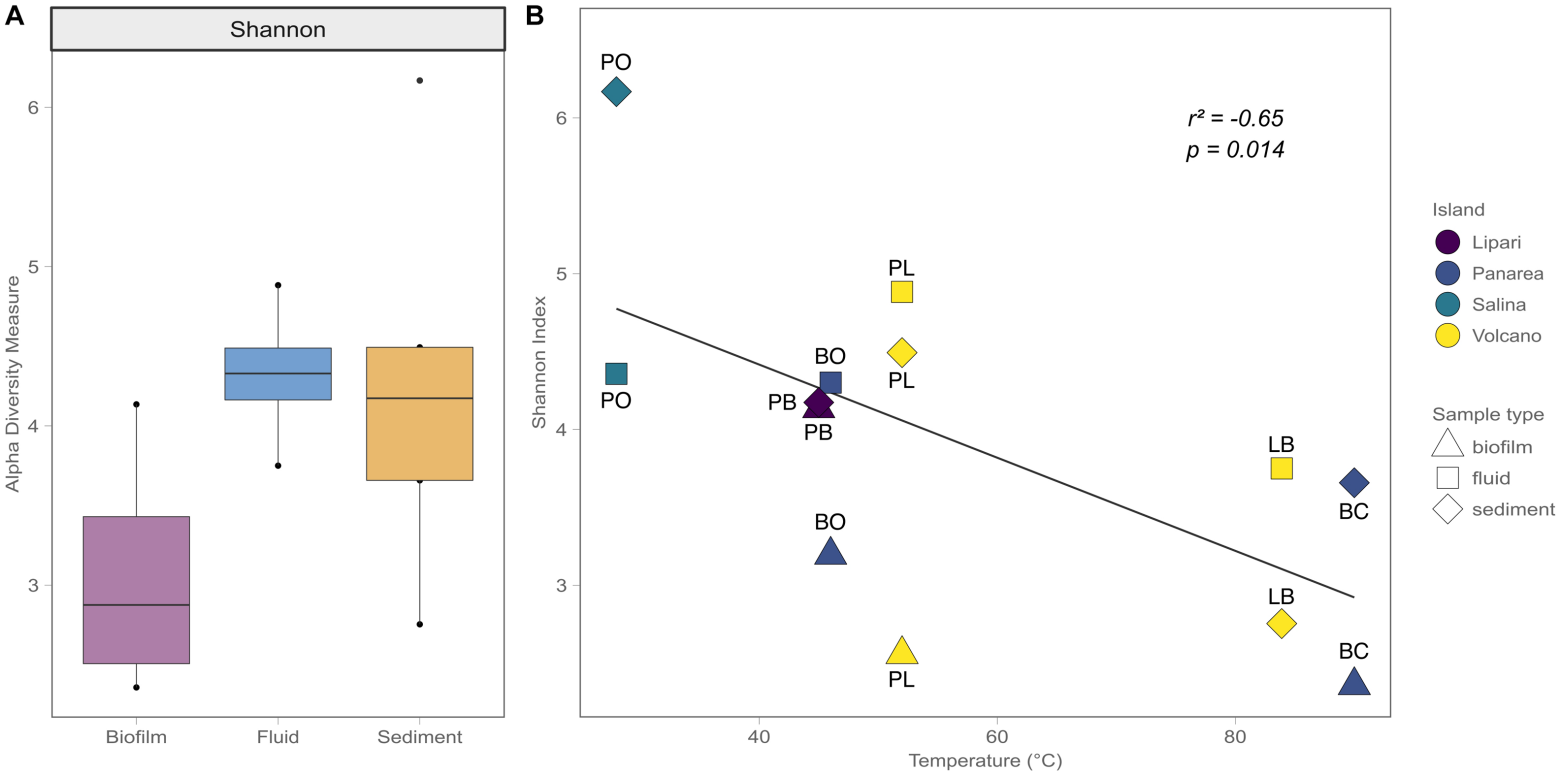
Shannon Alpha diversity indexes. **(A)** Alpha diversity measures in relation to the different sample types (Biofilm, Fluid, Sediment). **(B)** Alpha diversity measures in relation to the temperature, with the different Islands sampled, as well the different sample types.

The bacterial diversity of the shallow-water hydrothermal vents of the Aeolian archipelago was investigated using the V4-V5 variable region of the 16S rRNA gene. After guaranteeing high-quality data (quality check and data filtering steps), a total of 149,351 reads were obtained, and 2,145 ASVs identified. Regarding the alpha diversity of the different sample types, the biofilm samples had lower alpha diversity measure (3) in comparison with the fluid and sediment samples (4.3 and 4.2, respectively). In terms of variability, the fluid samples showed less sample variability (the difference between the lowest diversity value points to the highest) in relation to the biofilm and sediment samples, which could be related to the differences in the matrix of the different sample types (Figure 4A). For instance, deep geothermal fluids impose a stronger bottleneck in terms of possible redox couples and ecological niches to the microbial communities, compared to the sediment samples. Additionally, the temperature was found to be a major driver of diversity between samples (Pearson correlation test, *R*^2^ = 0.41), where higher temperature sites showed a decrease in alpha diversity (Figure 4B).

**Figure 5:**
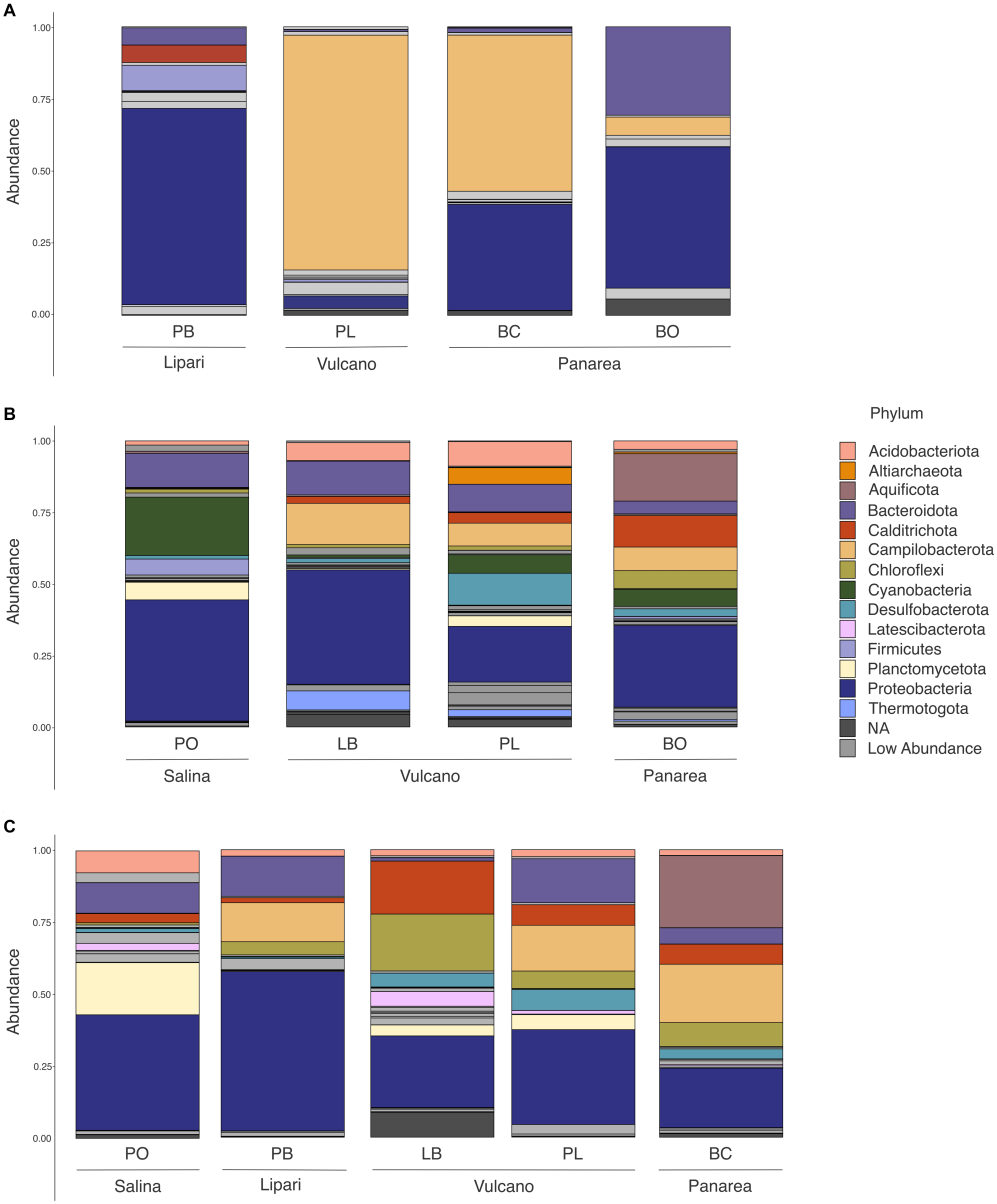
Phylum level abundance and distribution of the 16S rRNA diversity in all of the sample sites and sample types at the different islands of the Aeolian Archipelago. For simplicity, only abundances above 5% at each site are illustrated. **(A)** Biofilm samples; **(B)** Fluid samples; **(C)** Sediment samples.

The high-temperature sites, Baia Calcara and Levante Bay, characterized by temperatures above 80°C, were dominated by members belonging to Campylobacterota (formerly Epsilonproteobacteria), Proteobacteria, Aquificota, Bacteroidota, Chloroflexi and Acidobacteriota phylum (Figure 5). Among Campylobacterota, the order Campylobacterales was present in high abundances in all the high-temperature sites. Within Campylobacteriales, the genus *Nitratifactor* was only present at higher abundances (44%) in the biofilm sample of Baia Calcara, followed by *Sulfurimonas* (average abundance of 6.08% between the biofilm and sediment samples of Baia Calcara), *Campylobacter* (average abundance of 9% between the fluid and sediment sample of Levante Bay), and *Sulfurovum* genera (6.55% in the fluid sample of Levante Bay). The phylum Proteobacteria was composed entirely by class Gammaproteobacteria in the Baia Calcara site (average abundance of 12% between sediments and biofilm samples), and by Gammaproteobacteria and Alphaproteobacteria in the Levante Bay site (average abundance of 15% in both sample types and 8.5% in the fluid samples, respectively). Within Gammaproteobacteria, the orders Chromatiales and Thiomicrospirales were present in the highest abundances (average abundance of 24.6% at Levante Bay between the fluid and sediment samples, and 13.3% between the sediments and biofilm samples of Baia Calcara and the fluid samples of Levante Bay, respectively), with ASVs classified only to the genus *Candidatus thiobios* and *Thiomicrorhabdus*. The order Nitrococcales was present in lower abundances (average abundance of 6.1% between both sampling sites and all sample types, with the exception of the sediments of Levante Bay), and composed by the genus *Sulfurivirga*. Among Alphaproteobacteria, all ASVs were classified into the order Rhodobacterales in both the fluid and sediment samples of Levante Bay (average abundance of 8.5%), however, it was not possible to resolve them down to the genus level. The phylum Aquificota was composed entirely of members belonging to the order Hydrogenothermales, and the genus *Hydrogenothermus*, and only observed at the sediment samples of Baia Calcara (25.09%). Within Bacteroidota, only the order Flavobacteriales was identified in both the sediment and fluid samples at the Levante Bay site (average abundance of 9.3%), however, no ASVs were classified to the genus level. Acidobacteriota and Chloroflexi, albeit present in lower abundances (5.14% in sediment samples of Baia Calcara and 5.51% in fluid samples of Levante Bay, respectively), had as most abundant, members belonging to the order Thermoanaerobaculares, genus *subgroup* 23, and to the class *Anaerolineae*, respectively (Table 3).

**Table 3:** Taxonomic affiliation of the most abundant ASVs through SILVA database (to the lowest possible taxonomic resolution) and Ezbiocloud closest relative.

Sample type	SILVA classification	Ez-biocloud classification	Similarity (%)	Acc. no.
Biofilm	*Nitratifractor*	JQ611125_s	97.32	JQ611125
Biofilm	*Thiomicrorhabdus*	*Galenea microaerophila*	99.73	JQ080912
Biofilm	*Sulfurimonas*	JN873929_s	98.39	JN873929
Biofilm	*Sulfurivirga*	*Sulfurivirga caldicuralii*	99.73	jgi.1107633
Biofilm	Bacteria	JF320743_s	95.99	JF320743
Biofilm	*Rhodobacteraceae*	*Actibacterium pelagium*	*100*	NSBU01000007
Biofilm	*Arcobacteraceae*	AY922188_s	100	AY922188
Biofilm	*Flavobacteriaceae*	*Urechidicola croceu*	95.38	KX066850
Biofilm	*Colwellia*	*Colwellia rossensis*	100	U14581
Biofilm	SUP05_cluster	Maorithyas hadalis gill symbiont I	99.2	AB042413
Biofilm	*Nitrincolaceae*	AM402959_s	99.2	AM402959
Biofilm	Candidatus Nitrosopumilus	*Nitrosopumilus cobalaminigenes*	98.67	KX950757
Biofilm	*Sulfitobacter*	EU795102_s	100	EU795102
Biofilm	*Thiovulum*	DQ295692_s	92.25	DQ295692
Sediments	*Hydrogenothermus*	*Hydrogenothermus marinus*	99.2	AJ292525
Sediments	*Thiomicrorhabdus*	*Galenea microaerophila*	99.73	JQ080912
Sediments	*Sulfurivirga*	*Sulfurivirga caldicuralii*	99.73	jgi.1107633
Sediments	*Sulfurimonas*	JN873929_s	98.39	JN873929
Sediments	*Anaerolineae*	JF320743_s	96.52	JF320743
Sediments	Candidatus_Thiobios	AY310506_s	98.13	AY310506
Sediments	*Campylobacter*	JQ287068_s	98.12	JQ287068
Sediments	*Flavobacteriaceae*	*Urechidicola croceus*	95.38	KX066850
Sediments	*Rhodobacteraceae*	*Actibacterium pelagium*	100	NSBU01000007
Sediments	*Anaerolineaceae*	FJ905697_s	94.39	FJ905697
Sediments	*Mariprofundus*	*Mariprofundus aestuarium*	99.73	CP018799
Sediments	*Caldithrix*	*Caldithrix abyssi*	98.94	CM001402
Sediments	Bacteria	JF320743_s	95.99	JF320743
Sediments	*Caldithrix*	DQ925879_s	94.69	DQ925879
Sediments	*Woeseia*	JF344416_s	99.2	JF344416
Sediments	*Rhodothermaceae*	EU925913_s	99.19	EU925913
Sediments	*Rhodobacteraceae*	*Actibacterium pelagium*	100	NSBU01000007
Fluids	*Hydrogenothermus*	*Hydrogenothermus marinus*	99.2	AJ292525
Fluids	*Caldithrix*	*Caldithrix abyssi* DSM 13497(T)	98.94	CM001402
Fluids	*Synechococcus* CC99*02*	*Synechococcus sp.* CC9902	99.47	CP000097
Fluids	*Rhodobacteraceae*	*Actibacterium pelagium* JN33(T)	100	NSBU01000007
Fluids	Candidatus_Thiobios	AY310506_s	98.13	AY310506
Fluids	*Flavobacteriaceae*	*Urechidicola croceus* LPB0138(T)	95.38	KX066850
Fluids	*Sulfurovum*	HM591450_s	97.32	HM591450
Fluids	*Campylobacter*	JQ287068_s	98.12	JQ287068
Fluids	*Sulfurivirga*	*Sulfurivirga caldicuralii*	99.73	jgi.1107633
Fluids	*Thiomicrorhabdus*	*Galenea microaerophila*	99.73	JQ080912
Fluids	Subgroup_23	EU542513_s	98.39	EU542513
Fluids	Candidatus_Altiarchaeum	*Altarchaeum hamiconexum*	96.55	JAACVF010000098
Fluids	*Pseudoalteromonas*	*Pseudoalteromonas shioyasakiensi*s	100	AB720724
Fluids	NS5_marine_group	PAC001209_s	100	PAC001209
Fluids	*Anoxybacillu*s	*Anoxybacillus rupiensis*	100	AJ879076

Moderate-temperature sites, such as Bottaro, Pietra del Bagno, and Levante Port, characterized by temperatures ranging from 40°C to 53°C, presented a higher diversity of bacterial phyla. These sites were dominated by members belonging to Campylobacterota, Proteobacteria, Bacteroidota, Calditrichota, Chloroflexi, Cyanobacteria, as well as the Archaeal phylum Crenarchaeota and Altiarchaeota at low abundances. Among Campylobacteriota, the order Campylobacterales was only identified at the sediments and biofilm samples of the Levante Port site, with the highest abundant ASVs belonging to the family *Arcobacteraceae* (37.92% at the sediment samples), without classification to the genus level. Additionally, it was also possible to classify Campylobacterales into the genera *Thiovulum* and *Sulfurimonas* in the biofilm samples (23.40 and 19.40%, respectively), and *Sulfurovum* in the sediment samples (6.51%). Within Proteobacteria, the order Rhodobacterales (21.96% in biofilm sample at Bottaro and 5.70% in biofilm sample of Pietra del Bagno) was the most abundant group, however, it was not possible to classify them down to the genus level. Additionally, the family *Mariprofundaceae* and the assigned genus *Mariprofundus*, was the second most abundant genus within Proteobacteria (15.03%) and only present at the sediment samples of Pietra del Bagno. While in lower abundances, it was possible to identify the family *Thioprofundae*, composed by the genus *Thioprofundum* (7.47% in the sediment samples of Levante Port), and the family *Colwelliaceae*, where all the ASVs were assigned to the genus *Colwellia* (11.54% in biofilm sample of Pietra del Bagno). Moreover, it was also possible to identify the family *Thioglobaceae*, with the ASVs assigned to the clade SUP05 cluster (8.03% in the biofilm samples of Pietra del Bagno). Within the Bacteroidota, the order Flavobactereales constituted most of the ASVs (26.17% in the biofilm sample of Bottaro); however, classification could not be resolved to the genus level. Lower abundance ASVs belonging to Bacteroidota were also assigned to the order Ignobacteriales and the clade LheB3-7 (11.52% in sediment samples of Levante Port). The phyla Aquificota, similarly to the high-temperature sites, was composed entirely of ASVs belonging to the genus *Hydrogenothermus*, and only present at the fluid sample of Bottaro (16.57%). Among Calditrichota, the genus *Caldithrix* represented all the ASVs (17.32% in the sediment sample of Pietra del Bagno and 8.06% in the fluid sample of Bottaro). Additionally, the phototrophic bacterial phyla Cyanobacteria, was composed by the genus *Synechococcus* of the order Synechococcales in only the fluid samples (6.03 and 5.55% in Levante Port and Bottaro, respectively). The Archaeal phyla were detected in low abundances. Archaeal ASVs were classified into the genera *Candidatus_Nitrosopumilus* and *Candidatus_Altiarchaeum* (6.06% in the biofilm sample of Pietra del Bagno and 5.76% in the fluid sample of Levante Port, respectively). Interestingly, some lower abundance ASVs could not be classified up to the phylum level (8.59% in the sediment sample of Pietra del Bagno and 5.67% in the biofilm sample of Bottaro, respectively).

The lowest temperature site, Pollara, at the island of Salina, marked by temperatures of 28°C, was dominated by Cyanobacteria, Proteobacteria, Bacteroidota, and Firmicutes. The majority of the ASVs at lower temperatures were assigned to the phylum Cyanobacteria and the species *Synechococcus* sp. CC9902 (20.15% in the fluid sample). The order Alteromonadales with the genus *Pseudoalteromonas* (7.53% in the fluid sample), and the order Steroidobacterales, with the genus *Woesia* (9.15% in the sediment sample) were the most abundant within Proteobacteria. The phyla Bacteroidetes was composed of the orders Flavobacteriales and Rhodothermales (5.83% in the fluid sample and 5.06% in the sediment sample, respectively).

**Figure 6:**
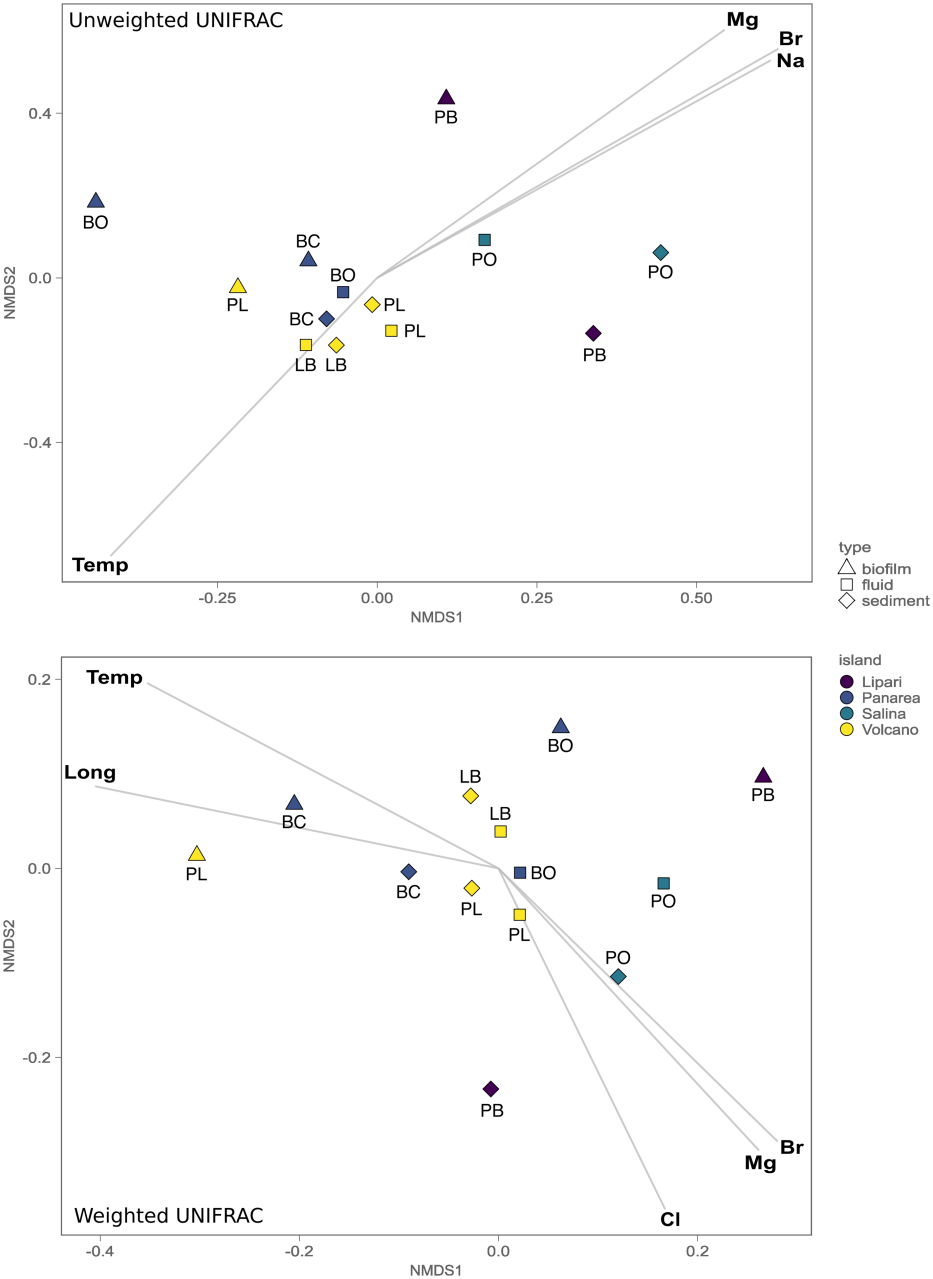
Non-metric multidimensional scaling (nMDS, stress 0.075) plot of the 16S gene amplicon microbial diversity based both the weighted and unweighted UniFrac measures, overlaid with environmental vector fitting, illustrating vectors with significant *p* values (*p* < 0.01).

To understand the role of the environmental parameters and the geochemistry in the structuring of the microbial communities inhabiting the SWHV of the Aeolian islands, we investigated the beta-diversity using both quantitative and qualitative approaches, more specifically, using weighted and unweighted UNIFRAC dissimilarity indexes, respectively, followed by environmental fitting. The unweighted UniFrac nMDS analysis shows a statistically significant separation between the communities of the islands of Lipari and Salina, and Vulcano and Panarea (ADONIS, *p* = 0.0016, *n* = 6). Additionally, the same separation was found between the composition of the communities of the biofilm samples, compared to the other sample types analyzed in the present study (ADONIS, *p* = 0.0075, *n* = 6). Through linear vector fitting versus the nMDS ordination, we observe which are the main parameters that better explain the microbial diversity of our samples (Figure 6). The group of Vulcano and Panarea can be explained by the temperature, and the group of Salina and Lipari, by the major cations and anions, Mg^2+^, Na^+^, and Br^−^. Using the weighted UniFrac, which weights the abundance of the taxa present, we found the same statistically significant separations between the same island groups (ADONIS, *p* = 0.0075, *n* = 6), and between biofilms and the other sample types analyzed in the present study (ADONIS, *p* = 0.0475, *n* = 6). However, we found differences regarding the main parameters explaining the abundance of microbial communities with vector fitting. For instance, here the biofilm samples of Levante Port and Baia Calcara were mainly explained by temperature and longitude. Additionally, the microbial diversity of Bottaro and Levante Port, in contrast with the unweighted version, are also explained by the major anions and cations, with the addition of Cl^−^.

## 4. Discussion

Shallow-water hydrothermal vents are marine environments associated with volcanic activity and tectonically active margins. Due to their unique characteristics, such as the presence of light, proximity to landmasses, and dynamic geochemistry, they harbor a highly diverse microbial consortia (Tarasov et al., 2005; Price and Giovannelli, 2017). The Aeolian archipelago is one of the most studied shallow-water hydrothermal systems in the world (Gugliandolo and Italiano, 1999; Simmons and Norris, 2002; Rogers and Amend, 2005; Rusch et al., 2005; Lentini et al., 2007, 2014; Manini et al., 2008; Maugeri et al., 2009, 2010, 2013; Gugliandolo et al., 2015; Gugliandolo and Maugeri, 2019). However, the role of the diverse geochemical environments found in the different islands in controlling the microbial community structure, is still poorly understood, and usually investigated in a handful of sites. To address this, we surveyed 6 different shallow water vents located in four different islands, combining 16S rRNA amplicon sequencing with geochemical and physico-chemical data. To the best of our knowledge, two of the investigated shallow vents (Pietra del Bagno and Pollara) were never reported before in the literature.

### 4.1. Geochemistry of the Aeolian shallow-water hydrothermal vents

Hydrothermal systems are characterized by the convective circulation of water that percolates into the crust, gets heated, and then is discharged at the sea floor. During this journey, the fluids are chemically and physically transformed by subsurface processes, such as temperature-dependent water-rock reactions, leaching of transition metals and volatiles from the host lithologies, and phase-separation events (Fournier, 2007; German and Seyfried, 2014). These processes, although occurring in a dynamic continuum, are generally separated into three distinct areas: the recharge zone, the reaction zone, and the upflow zone. Due to the inherent characteristics of the locations within the subsurface where they take place, these areas are marked by the occurrence of different processes. The recharge zone is characterized by the fixation of alkali metals, boron, and the fixation of Mg^2+^ associated with the mineralization of smectite and chlorite. Deep in the system (reaction zone), anorthite is transformed to albite (albitization), resulting in the exchange of Na^+^ and Si^4+^ for Ca^+^ into the fluids, as well as metal mobilization. Additionally, the high temperatures and pressures present at depth allow phase separation events, separating the low weight ions (migrate to the vapor phase) from the heavier weight ions (remain in the fluids). Cl^−^, by remaining in the fluid phase, modifies the salinity content of the hydrothermal fluids. In the upflow zone, further low-temperature water-rock reactions can occur, such as the precipitation of anhydrite and other minerals (Berndt et al., 1988; Tivey, 2007; Alt, 2013; German and Seyfried, 2014). During ascent, the fluids can either be channeled, feeding directly from deep subsurface reservoirs, or diffuse, with significant mixing of end member fluids with seawater prior to discharge. All together these processes entail different ratios of mixing with seawater, contributing to additional alterations to the fluids before they are discharged at the seafloor where they are collected (Price and Giovannelli, 2017). By analyzing the enrichment and depletion of major ions and cations in vent fluids, we can have a deeper understanding of the main processes occurring in the subsurface. These geochemical processes define the geochemical space available for microbial communities to thrive in these ecosystems, and are fundamental in investigating the microbial diversity of shallow-water hydrothermal vents (Price et al., 2015; Price and Giovannelli, 2017).

In the present study, we leverage the information available in the already published literature on the geochemistry of hydrothermal fluids of the shallow vents of Vulcano, Panarea, and Stromboli (Figure 3) combined with new data. Furthermore, we add for the first time geochemical data for the shallow vents of Lipari and Salina (Pietra del Bagno and Pollara, respectively). It is important to note that subsurface processes at shallow-water hydrothermal vents hosted in volcanic arc settings have not been characterized to the same level of detail as Mid-Ocean Ridge hosted deep-sea systems. For this reason, our study relies on previous characterization of Panarea fluids by Price et al. (2015) and conceptually extended to the other shallow vents investigated. By combining published and new geochemical data we identified three mixing trends (Type 1, Type 2, and Type 3) already described for Panarea by Price et al. (2015).

The observed different trends reveal that these hydrothermal fluids evolved differently, and therefore were subjected to different subsurface processes within the Panarea hydrothermal system. Data from Vulcano, Lipari, and Salina shallow vents shows that fluids from diverse islands fall within the Type 3 trend (Figure 3). These fluids do not show enrichments of major anions and cations, but instead, show depletions of the major species. The evolution of Type 3 fluids results in acidic fluids, with concentrations of major ions similar to sea-water concentrations, whereas type 1 and type 2 fluids are the result of deep recirculating brine and focused upflow of shallower brines, respectively. This suggests that the difference between these fluid types is directly related to subsurface processes, such as phase-separation deep in the system, the scrubbing of exsoluted gasses that percolate up the crust, as well as the mixing of the diffuse flow fluids with sea-water (Price et al., 2015). Based on our data, it appears that the evolution of Type 3 fluids might be a common process in the shallow water vents of the Aeolian Archipelago. Additionally, and as evident in Price et al. (2015), most of the fluids, including the ones in the present study, do not extrapolate to zero in both SO_4_^2−^ and Mg^2+^. This can be related to seawater mixing as the fluids approach the surface, due to their diffuse nature. However, in the Bottaro site, the fluids are enriched in Mg^2+^. It was hypothesized that Mg^2+^ enrichments in Panarea could be related to the leaching of host rocks due to acidification, by means of argillic alteration (Reeves et al., 2011). Since volcanic arc hydrothermal systems are not exposed to the same pressures compared to their deep-sea counterparts (Price and Giovannelli, 2017), the presence of free gas phases may also be contributing to the depletion of Na^+^ and Cl^−^ observed relative to sea water (Figure 3), altering the salinities of the fluids as they percolate through the hydrothermal system. All the fluid samples collected in our study had Br^−^/Cl^−^ ratios lower than seawater, suggesting the presence of dissolved halite in solution. Albitization, which occurs deep in the system and results in the release of Ca^+^ in exchange for Na^+^ (Alt, 2013), does not seem to be a major process affecting the evolution of our fluid samples, compared to other fluids recovered from Panarea and Vulcano (Figure 3).

While the geochemical composition of the fluids is an important parameter in controlling the diversity of microbial communities, the availability of organic carbon for heterotrophic consumption is fundamental in driving community composition. In hydrothermal systems organic carbon can either be delivered through the subsurface fluids as dissolved organic carbon produced or recycled at depth (Fullerton et al., 2021), or from the water column, either as sinking organic matter particles, lateral advections of surface primary productivity, and terrigenous run offs (Gomez-Saez et al., 2015; Price and Giovannelli, 2017; LaRowe et al., 2020). Additionally, it could be directly produced *in situ* by a diverse community of phototrophic and chemolithotrophic microorganisms (Price and Giovannelli, 2017). The organic carbon delivered to the system is accumulated in the sediments, and analyzing the sedimentary organic matter content can provide an estimate of the organic load on the vent system. In our data the sedimentary organic matter, measured as biopolymeric organic carbon (BPC) was highly variable between the different sites, and did not show linear relationships with temperature, in contrast to what has been observed in a previous study on a shallow-water hydrothermal vent system (Giovannelli et al., 2013; Figure 2A). The lowest amounts of BPC was measured in the site with the lowest temperatures (Pollara, 28°C and Pietra del Bagno, 45°C), and could be related to a to hydrodynamic processes, perturbing the system in these specific locations (Bao et al., 2018), as well as to the fact that these sites are further away from the coast, and thus representing lower amounts of terrigenous inputs to the system. The high concentrations found in Bottaro are instead related to the thick microbial biofilms attached to the sand and gravel. The concentration of chlorophyll-*a* (chl-*a*) pigments was lower at the higher temperatures sites (>80°C; Figure 2B) in line with previous observations (Giovannelli et al., 2013). The presence of Chl-*a* in the sediments can be either related to input of fresh organic carbon from the water column or to the presence of phototrophs in the sediments and biofilms of the vent. The presence of known phototrophs in the sediment and biofilm samples (see below) suggests that the high chlorophyll concentrations found in the same sites are the results of *in situ* production, possibly taking advantage of the nutrients and CO_2_ released by the shallow vents. Additionally, the chl-*a* values were higher at the Levante Port and Bottaro sites, which had similar depths (average 6.4 meters), suggesting that beside temperature, the depth of the shallow vent could play a role in controlling the presence and abundance of photosynthetic organisms.

### 4.2. Microbial diversity

Previous studies conducted at shallow-water hydrothermal vents have demonstrated that a highly diverse microbial consortia inhabit these ecosystems (Hoaki et al., 1995; Sievert et al., 2000; Manini et al., 2008; Maugeri et al., 2009, 2010; Zhang et al., 2012; Price et al., 2013; Kerfahi et al., 2014; Lentini et al., 2014; Dávila-Ramos et al., 2015; Gugliandolo et al., 2015; Gomez-Saez et al., 2017). In the present study, we found that the microbial communities of the Aeolian Islands shallow-water vents are dominated by members belonging to diverse phyla, such as Proteobacteria, Campylobacteriota, Bacteroidota, Cyanobacteria, Chloroflexi, among others present at lower abundances. The major lineages found here have been reported in other shallow-water vents (Maugeri et al., 2010; Zhang et al., 2012; Kerfahi et al., 2014; Lentini et al., 2014; Dávila-Ramos et al., 2015; Gugliandolo et al., 2015; Gomez-Saez et al., 2017; Price and Giovannelli, 2017), illustrating their important ecological role in the functioning of the shallow-water vent ecosystem.

Our data shows that temperature is a major driver explaining the microbial diversity (alpha-diversity, Pearson correlation test, *R*^2^ = 0.41, *p* < 0.05). High-temperature sites (>80°C) were characterized by lower diversity in comparison to lower and moderate temperatures sites (28°C to 50°C; Figure 4B). Temperature has been shown to be one of the major factors controlling microbial diversity in a number of diverse geothermal ecosystems, including shallow-water vent environments (Giovannelli et al., 2013; Gugliandolo et al., 2015), deep-sea hydrothermal vents (Dick et al., 2013; Ding et al., 2017), and hot springs (Chan et al., 2017; Fullerton et al., 2021).

High-temperature locations, such as Baia Calcara and Levante Bay sites in the islands of Vulcano and Panarea, respectively, were characterized by temperatures above 80°C. The microbial communities inhabiting these high-temperature shallow vents were dominated by groups related to known chemolithoautotrophic, thermophilic bacteria, harnessing energy through the oxidation of reduced sulfur species and hydrogen. These types of metabolic strategies have been widely reported in shallow-water, deep-sea hydrothermal vents, as well as hot-springs (Tarasov et al., 2005; Yamamoto and Takai, 2011; Inskeep et al., 2013; Price and Giovannelli, 2017). The Baia Calcara and Levante Bay sites had as most abundant, ASVs related to the genera *Nitratifactor*, *Thiomicroarhdbus*, *Hydrogenothermus*, *Candidatus_thiobios,* and the family *Rhodobacterecea*e. The biofilm community at the Baia Calcara site was mostly composed of members belonging to *Nitratifactor* and *Thiomicroarhdbus*. *Nitratifactor* has been previously described in shallow and deep-sea hydrothermal systems (Zhang et al., 2012; Zeng et al., 2021). The closest sequence to our ASVs was recovered from a shallow-water hydrothermal system in Kueishan Island, Taiwan (unpublished data). Interestingly, this group was not present in the sediment samples of the same site. Additionally, the genus *Thiomicroarhdbus* was also present in high abundances in the biofilm samples, and in lower abundance also in the sediment sample. It was possible to assign this ASVs to the species *Galenea microaerophila* (99.73% similarity) (Giovannelli et al., 2012). Initially described from a shallow-water vent from Paleochori Bay, on the island of Milos, *Galenea microaerophila* grows chemolithoautotrophically, through the oxidation of thiosulfate as electron donor, and oxygen (5%) as electron acceptor (Giovannelli et al., 2012). The sediment sample at Baia Calcara was mostly composed by the genus *Hydrogenothermus*, of the phylum Aquificota. This ancestral and deep-branching bacterial phylum is highly prevalent in geothermal environments (Hedlund et al., 2015; Giovannelli et al., 2017), and it is capable of fixing CO_2_ into biomass using energy derived from H_2_S and H_2_ with either elemental sulfur, nitrate or oxygen (at low partial pressures) as electron acceptor. The dominant ASV in our dataset was related to the species *Hydrogenothermus marinus* (99.2% similarity), a hydrogen-oxidizing, chemolithoautotrophic, thermophilic bacterium (Stöhr et al., 2001) originally isolated from sediment samples from Vulcano island.

At the fluid and sediment samples at Levante Bay, the other high temperature site, the family *Rhodobactereceae* and the genus *Candidatus* thiobios were present in highest abundances. The family Rhodobactereceae is widespread in marine environments, and are capable of growing through the utilization of multiple organic, as well as inorganic compounds, contributing to their vast ecological diversity (Pohlner et al., 2019). In our study, it could also be identified in moderate to lower temperature sites. The ASVs classified as *Rhodobacterecea*e were further related to the species *Actibacterium pelagium* (100% similarity), which have been isolated from the seawater of the Mediterranean Sea, Pacific and Atlantic Oceans, growing heterotrophically on a variety of organic carbon sources (Guo et al., 2017). Even though members belonging to *Rhodobactereceae* have been previously identified in other hydrothermal systems (Forget et al., 2010; Zhang et al., 2012; Pohlner et al., 2019), to our knowledge, this is the first time this species has been reported in a hydrothermal vent environment. The genus *Candidatus* thiobios is associated with ectosymbiotic bacteria growing on the marie ciliate *Zoothamnium niveum*, commonly found in sulfide-rich habits, including hydrothermal vents (Rinke et al., 2006; Bright et al., 2014). The presence of high abundance of this genus could be related to the presence of the *Zoothamnium niveum* inhabiting this specific site.

Both sites (Baia Calcara and Levante Bay), included lower abundances of sequences assigned to the genus *Sulfurimonas*, *Sulfurivirga*, *Sulforovum*, and *Campylobacter*, all belonging to the Campylobacterota phylum (formerly known as Epsilonproteobacteria). Members of the *Sulfurimonas* genus are widespread in sulfidic environments and are capable of using multiple reduced sulfur species as electron donors, such as thiosulfate, hydrogen sulfide, and elemental sulfur (Han and Perner, 2015). The genus *Sulfurivirga* could be assigned to the species *Sulfurivirga caldiculari* (99.73% similarity), a thiosulfate-oxidizing, thermophilic, chemolithoautotrophic bacterium, described in a shallow-water vent system in Japan (Takai et al., 2006). Similarly, *Sulfurovum* is also associated with thiosulfate oxidation and chemolithoautotrophy (Inagaki et al., 2004). *Campylobacte*r, within Campylobacteriota, is ubiquitous in hydrothermal environments, including shallow-water hydrothermal vents (Rinke et al., 2006; Anderson et al., 2011; Giovannelli and Vetriani, 2017; Wang et al., 2017). These genera have been associated with high-temperature high sulfidic locations in other shallow-water, as well as deep-sea hydrothermal systems (Forget et al., 2010; Zhang et al., 2012; Giovannelli et al., 2013; Gugliandolo et al., 2015; Gomez-Saez et al., 2017; Price and Giovannelli, 2017). Members of the Campylobacterota are present in most sites presenting higher temperatures (Levante Bay, Levante Port, Bottaro, Baia Calcara and Pietra del Bagno) and dominate the biofilm communities of Levante Port and Baia Calcara. Hydrothermal vents are often enriched in reduced sulfur species (German and Seyfried, 2014; Price and Giovannelli, 2017). The presence of sulfur-related metabolisms, coupled to inorganic carbon fixation, highlights the importance of these types of metabolic strategies for the function of hydrothermal systems, as well as the importance of these ecosystems to mediate the biogeochemical cycles of sulfur and carbon. Additionally, these high temperature locations had the highest protein/carbohydrate ratios (15.2 in Baia Calcara and 12.6 in Levante Port), suggesting high *in situ* productivity. Since at these locations, recovered ASVs are related to known chemolithoautotrophic groups, indicates that they are major contributors to primary productivity in high temperature shallow water hydrothermal vents. Similarly, Gomez-Saez et al. (2017), reported that chemoautotrophy constitutes an important contribution to fresh organic matter production, accounting for up to 65% of autotrophic carbon fixation in the shallow-water hydrothermal vents of Dominica.

At moderate to low temperatures an increase in phototrophic microbial lineages was observed, also reflected in the quantities of BPC and Chlorophyll-*a*. The prevalent photosynthetic group observed in this study was assigned to the genus *Synechococcus* and to the species *Synechococcus* sp. CC9902 (99.47% similarity), belonging to the phylum Cyanobacteria. The presence of phototrophs in shallow-water hydrothermal systems is well documented, as well as a key feature of these systems (Tarasov et al., 1990, 1999, 2005; Sievert et al., 2000; Zhang et al., 2012; Maugeri et al., 2013; Price et al., 2013; Lentini et al., 2014; Dávila-Ramos et al., 2015; Gugliandolo et al., 2015; Gomez-Saez et al., 2017; Price and Giovannelli, 2017). Here, *Synechococcus* sp. CC9902 was present in lower abundances at moderate temperatures in Bottaro and Levante Port, and high abundances in Pollara, the lowest temperature site. *Synechococcus* sp. CC9902 is widespread in the oceans and is described as having increased plasticity to diverse saline concentrations and light intensities (Ludwig and Bryant, 2012; Kim et al., 2018). Furthermore, the presence of this taxon has already been described in shallow-water hydrothermal vent environments (Maugeri et al., 2013). However, in other shallow-water vents systems, the specific lineages within Cyanobacteria differ. This suggests that in shallow-water hydrothermal vents the photoautotrophic niche space can be occupied by diverse lineages of phototrophic taxa. Notwithstanding, it is important to note that, even though the presence of Cyanobacteria in shallow water hydrothermal vents is well documented, the possibility of mixing with background seawater cannot be discarded. For instance, it has been shown that fluid mixing with seawater could be the result of the convective cells in porous media (Yücel et al., 2013).

The shallow water vent Pietra del Bagno, Lipari, due to the presence of orange precipitates surrounding the venting site, was deemed to be iron-rich. The sediment community was mainly composed by the family *Anaerolineares* of the phylum Chloroflexi, and the genus *Mariprofundus* belonging to Proteobacteria. Members belonging to Chloroflexi are ecologically and physiologically diverse, allowing them to colonize a wide variety of environments (Ward et al., 2018; Vuillemin et al., 2020). Despite being originally associated with anoxygenic phototrophy, they are also known to employ other metabolic strategies (McIlroy et al., 2017). The family *Anaerolineares* is associated with hydrocarbon oxidation and is known to have important roles in the functioning of anaerobic digestion systems (McIlroy et al., 2017). Nonetheless, they have been reported in deep-sea and shallow-water hydrothermal vents (Rajasabapathy et al., 2018; McGonigle et al., 2020; Wang et al., 2020). The closest relative to our sequences was reported from Fe-rich hydrothermal sediments of two submarine volcanoes of the Tonga volcanic arc (Forget et al., 2010). Interestingly, this bacterial family has also been observed to be present in high abundances in Fe-enriched locations (Hoshino et al., 2016; Ward et al., 2017). Other members of Chloroflexi, such as *Ardenticatena maritima*, grow by dissimilatory iron-reduction (Kawaichi et al., 2013). Thus, it is possible that *Anaerolineares* can couple the oxidation of organic matter to iron-reduction, making them major players in the biomineralization of organic matter in shallow-water vent environments.

The genus *Mariprofundus* of Zetaproteobacteria are well-known iron(II)-oxidizers that couple the iron (II) oxidation to the reduction of oxygen or nitrate (Chiu et al., 2017; McAllister et al., 2019). Members belonging to the phyla Zetaproteobacteria are widespread in hydrothermal Fe-rich environments (Emerson and Moyer, 2002; Emerson et al., 2010; Forget et al., 2010; Gomez-Saez et al., 2017; McAllister et al., 2019). Within *Mariprofundus*, it was possible to assign to the species *Mariprofundus aestuarium* (99.73% similarity), previously isolated from redox-stratified water columns, and capable of growing through the oxidation of iron (II) (Chiu et al., 2017). Additionally, albeit in lower abundances, it was possible to identify the genus *Caldithrix*. More specifically, we could assign our sequences to the species *Caldithrix abyssi* (98.94% similarity). This species is a known chemolithoautotroph, capable of growing anaerobically through the reduction of nitrate, using hydrogen as an electron donor (Miroshnichenko et al., 2003). Our geochemical analysis did not identify nitrate in the fluids at this site (data not shown); however, it was recently found that an environmentally assembled genome (MAG) of this species codes for a homolog of an extracellular respiratory system found in *Geobacter*, associated with iron reduction (Fortney et al., 2018). Given that this terminal electron acceptor was not tested in the original isolate (Miroshnichenko et al., 2003), the coupling of hydrogen oxidation to iron reduction could potentially suggest a metabolic strategy employed by *Caldithrix*. Notably, previous studies conducted at Fe-rich shallow-water vents have reported similar communities to the ones observed at this site (Handley et al., 2010; Meyer-Dombard et al., 2013; Gomez-Saez et al., 2017). Thus, these lineages can be considered to be important in driving the iron cycling in the shallow-water vent ecosystem, making important contributions to the biogeochemical cycles of iron. Interestingly, it was not possible to identify iron oxidizers in the biofilm community in high abundance at the same site. Conversely, the genus *Collwelia* was present in high abundances, followed by the clade SUP05. Members belonging to *Collwelia* are psychrophilic, heterotrophic bacteria, ubiquitous in cold marine environments (Techtmann et al., 2016), including sea ice (Zhang et al., 2008), polar sediments (Wang et al., 2015), and deep-sea trenches (Nogi et al., 2004). Specifically, we identified the species *Colwellia rossensis* (100% similarity), isolated from coastal Antarctic sea ice (Bowman et al., 1998). Colwellia species have been isolated from marine environments, and are characteristically psychrophiles (Bowman et al., 1998). The presence of ASVs related to this species at higher temperatures suggests a bigger temperature gradient to which this group is adapted to. The clade SUP05 is composed of known sulfur-oxidizing bacteria, also described as having a heterotrophic-based metabolism (Spietz et al., 2019; Morris and Spietz, 2022). At this location, the protein to carbohydrate ratios is 1.13. As reported by Pusceddu et al. (2003), since proteins are readily mobilized, as compared to carbohydrates, low ratios suggest the presence of aged organic matter (not freshly produced), and probably indicates external input as a potential source of organic matter to the biofilm communities.

Additionally, in moderate temperatures sites, it was possible to identify in higher abundance the families *Flavobacterecea*e and *Arcobactereceae*, in the biofilms of Bottaro and Levante Port, and the genus *IheB3-7* at the sediment sample of Levante Port. The family *Flavobacteriaceae* of the Phylum Bacteroidetes, ubiquitous in marine environments, are known to degrade complex hydrocarbons, with large numbers of glycosyl hydrolases and peptidases encoded in the genomes of this bacterial group (Gavriilidou et al., 2020). Moreover, the family *Arcobactereceae*, also widely distributed in marine environments (Venâncio et al., 2022), has been reported in shallow vent hydrothermal environments (Maugeri et al., 2009; Gugliandolo et al., 2015; Gomez-Saez et al., 2017).

At the lowest temperature site (Pollara), Cyanobacteria was the most abundant group, and with lower abundances, the genera *Pseudoalteromonas*, *Woesia*, NS5 marine group, and *Anoxybacillus*. These microbial groups are associated with heterotrophic metabolisms, and given the protein/carbohydrate ratios at this location of 3.64, as well as Chla-a concentration of 1.0 μg g^−1^, suggests that these groups rely on the export of organic carbon from the water column into the vent system, coupled with the usage of aged organic matter derived from external input, and *in situ* production from phototrophic organisms. *Pseudoalteromonas*, *Woesia*, and NS5 marine group are widely distributed in marine environments (Holmström and Kjelleberg, 1999; Du et al., 2016; Priest et al., 2022). Additionally, *Anoxybacillus* was also reported in deep-sea hydrothermal vent sediments (Cheng et al., 2021). Here, it was possible to assign *Anoxybacillus* to the species *Anoxybacillus rapiensis* (100% similarity), a thermophilic, anaerobic, heterotrophic bacterium, isolated from diverse hot springs in Bulgaria (Derekova et al., 2007).

While temperature is one of the key variables controlling the microbial diversity in our dataset, other environmental parameters contribute significantly in shaping microbial communities of the different sample types collected (fluids, sediments and biofilms) across the geochemically diverse vents sampled. This has been recently demonstrated in hot springs (Fullerton et al., 2021), where a combination of the geological settings, the aqueous geochemistry and trace element availability provided strong explanatory power of the beta diversity turnover among the sampled springs. In this work unweighted and weighted UniFrac based multivariate analysis (Figure 6) revealed the presence of three statistically significant groups. The first was composed of the biofilm samples, where no significant correlations with the measured parameters were found to significantly explain the diversity. The biofilm composition and architecture are found to be influenced by both deterministic, as well as stochastic effects. For instance, previous studies have shown that biofilm thickness drives the biofilm community and diversity (Suarez et al., 2019), combined with the age of the biofilm and other environmental variables. The second major group, encompassing the samples of fluids and sediments from the islands Vulcano and Panarea, could be explained by the temperature. As discussed above, these locations were marked by moderate to high temperatures, with ASVs associated with microbial lineages related to the oxidation of hydrogen or reduced sulfur species. In hydrothermal systems fluid ascent is facilitated by the permeability of lithospheric fault systems, allowing a more focused channeling of the fluids to the surface (Pearce et al., 2019). Moreover, hydrothermal systems further away from fault systems are likely to show weak thermal anomalies (Taillefer et al., 2018). Consistent with this, the site with the lowest temperature, Pollara in Salina, is the sampling site further away from known fault systems in the area. Interestingly, we found that temperature and Mg^2+^ follow opposite correlation patterns. Magnesium is depleted in end-member hydrothermal fluids, resulting from water-rock reactions during the circulation of the fluids in the hydrothermal system (Alt, 2013). The presence of higher quantities of Mg^2+^ in low temperature sites, can entail less degree of water-reactions, which could be a function of the weak thermal anomalies found in the island of Salina and Lipari. Consistent with this, these islands present less geothermal activity compared to the other island samples in this study.

In the third group, encompassing the sites from the islands of Salina and Lipari, the microbial diversity was explained by the major anions and cations Mg^2+^, Br^−^, and Na^+^. Despite the geochemical analysis encompassing our samples in combination with the literature available placed our samples within Type 3 fluids, small variations of the major anions and cations show statistically significant correlation with the microbial distribution in both these islands. Diverse subsurface processes could result in similar concentrations of the major anions and cations, but result in a differential enrichment of minor elements, such as trace elements (Rollinson and Pease, 2021). Recent studies have shown that trace elements play key roles in shaping microbial niche occupation, as well as have been used to improve the cultivation of extremophilic organisms from natural hot springs (Meyer-Dombard et al., 2012; Shafiee et al., 2021).

Similarly to the unweighted UniFrac, with the weighted version, which weights the abundance of the taxa present, we found the same statistically significant separation between the communities of Vulcano/Panarea and Salina/Lipari, as well as the biofilm samples and the other samples types. However, conversely to the unweighted UniFrac, we found the temperature to be correlated with the abundance of taxa of Baia Calcara and Levante Port biofilm communities. These were dominated by thermophilic communities belonging to *Nitratifractor* and *Thiomicrorhabdus*. Therefore, both these parameters can act as strong selective pressures, driving not the structuring of the biofilm communities of Baia Calcara and Levante Port, but selecting for thermophilic, chemolithoautotrophic groups. The correlation with longitude could be related with the location of the islands within the Aeolian volcanic arc. It has been previously demonstrated that the magmatic composition of the Aeolian arc exhibits within-islands variations (Peccerillo et al., 2013). These differences could entail differential inputs of magmatic volatiles into the hydrothermal fluids, which in turn could be influencing the microbial communities inhabiting the shallow-water hydrothermal vents. It was also possible to find the major elements correlating with the abundance of the communities of the islands of Lipara and Salina, and with weighted UniFrac, the communities of Vulcano and Salina.

In the Aeolian Archipelago it is possible to find a highly diverse set of marine habitats. These habitats, in turn, harbor an abundant number of species, some of which with endangered status (Álvarez et al., 2019). For this reason, over the years, there have been increasing efforts to enhance the conservation measures on the Aeolian Islands, through the practices of sustainable fishing, tourism, and community education (Álvarez et al., 2019). The presence of high hydrothermal activity should further augment the need for conservation practices in these islands, for different reasons. One is that the microbial communities inhabiting these vents play important ecological roles for the entire shallow marine ecosystem (Tarasov et al., 2005; Price and Giovannelli, 2017), potentially sustaining primary productivity in surrounding ecosystems (Price and Giovannelli, 2017). Given their key roles as primary producers, these microbial communities sustain complex food webs on the surrounding islands. Secondly, extreme environments are often looked upon for the isolation of microorganisms that produce industrially and medically relevant metabolites, such as novel enzymes and antibiotics (Littlechild, 2015; Sayed et al., 2020; Petrillo et al., 2021). For instance, novel enzyme-producing Bacillus have been isolated from the Island of Panarea, whereas novel hydrolases have been identified on environmental DNA libraries from the sediments of the shallow-vents of the island of Vulcano (Lentini et al., 2007; Gugliandolo et al., 2012; Placido et al., 2015). Thirdly, in recent years there have been efforts to apply conservation status to deep-sea hydrothermal vents (Van Dover, 2012; Van Dover et al., 2018). We argue that shallow-water hydrothermal vents play an equally important role in the functioning of marine ecosystems, and should be explicitly acknowledged in marine spatial planning and conservation efforts, not only because of their uniqueness, but because they represent an accessible window for us to appreciate the intricate relationships between the geosphere and biosphere.

## 5. Conclusion

In conclusion, our study shows that the Aeolian archipelago shallow water hydrothermal vents harbors a highly diverse microbial consortia. In line with previous studies conducted in these unique ecosystems, we found the presence of ASVs related to organisms employing diverse metabolic strategies, ranging from chemolithoautotrophy, heterotrophy and phototrophy. Furthermore, we found those same groups to be correlated strongly with temperature, where we could observe a transition from higher temperatures (mainly ASVs related to chemolithoautotrophic groups) to moderate and lower temperatures (mainly ASVs related to phototrophic and heterotrophic groups). This transition is commonly observed in geothermal environments, and can be considered as a definite characteristic of these systems. Furthermore, through the analysis of BPC, we found that different types of organic matter are available to the resident communities, from terrigenous input exported to the system, as well as *in situ* production by chemolithoautotrophic and phototrophic groups. This supports the literature on how shallow water hydrothermal vents are considered high energy environments. Through beta diversity analysis, we found the presence of three significant groups, composed of the biofilm samples, and the sediment and fluid samples of the islands of Vulcano and Panarea, and Lipari and Salina. The group of Vulcano and Panarea was correlated with temperature, as these locations were marked by moderate to high temperatures, and they were dominated by ASVs related to chemolithoautotrophic groups. As for the group of Lipari and Salina, the distribution of the microbial communities was correlated with major ions. This suggests that subsurface water rock reactions have an influence on the concentration of the major ions, which in turn, can influence the microbial communities inhabiting these shallow water hydrothermal vents. Additionally, we found that the longitude, together with the temperature, was a significant vector in the weighted UNIFRAC. This correlation could be due to the location of the islands within the Aeolian volcanic arc and the different geothermal activity within each island.

Additionally, our compilation of fluid data from previous studies showed the same trend noted by Price et al. (2015), wherein in these hydrothermal systems, one can find 3 types of fluid evolution, showing differential enrichments and depletions of the major anions and cations, and therefore hinting at different subsurface processes taking place in the different islands. Given that the Aeolian archipelago shallow water hydrothermal vents, and the microbial communities inhabiting them, have important ecological roles for the surrounding marine environment, we think it is important their inclusion into present and future conservation efforts. Finally, shallow-water vents and the volcanic arc lithologies at which they occur are still understudied, compared to the basaltic, mid-ocean ridge lithologies from their deep-sea counter-parts. Our study is the first stage of a deeper characterization of the Aeolian archipelago shallow-water systems, and future studies will shed light on the ecological role of the communities in these systems, as well as on the local biogeochemical cycles.

## Data availability statement

The datasets presented in this study can be found in online repositories. The names of the repository/repositories and accession number(s) can be found below: https://www.ebi.ac.uk/ena, PRJEB54611, https://zenodo.org/record/7480614.

## Author contributions

BB carried out data analysis, geochemical experiments and wrote the first draft of the manuscript. BB, AF, MS, MG, AB, MCO, and LD produced the geochemical and microbiological data. MS and AF carried out Dada2 and Phyloseq and statistical analysis. AF, AC, MC, GB, RP, MI, CV, and DG planned the expedition and collected the samples. RP, CV, AC, and DG supervised the study. All authors contributed equally to the final version of the manuscript.

## Funding

This work is partially funded by the European Research Council (ERC) under the European Union’s Horizon 2020 research and innovation program (grant agreement No. 948972) to DG. Partial funding was from the NASA Habitable Worlds program under grant 80NSSC20K0228 to REP.

## Conflict of interest

The authors declare that the research was conducted in the absence of any commercial or financial relationships that could be construed as a potential conflict of interest.

## Publisher’s note

All claims expressed in this article are solely those of the authors and do not necessarily represent those of their affiliated organizations, or those of the publisher, the editors and the reviewers. Any product that may be evaluated in this article, or claim that may be made by its manufacturer, is not guaranteed or endorsed by the publisher.

## Footnotes

^1^https://imr.bio

^2^www.arb-silva.de
